# A distinct and active bacterial community in cold oxygenated fluids circulating beneath the western flank of the Mid-Atlantic ridge

**DOI:** 10.1038/srep22541

**Published:** 2016-03-03

**Authors:** Julie L. Meyer, Ulrike Jaekel, Benjamin J. Tully, Brian T. Glazer, C. Geoffrey Wheat, Huei-Ting Lin, Chih-Chiang Hsieh, James P. Cowen, Samuel M. Hulme, Peter R. Girguis, Julie A. Huber

**Affiliations:** 1 Josephine Bay Paul Center, Marine Biological Laboratory, 7 MBL St., Woods Hole, MA 02543; 2 Department of Organismic and Evolutionary Biology, Harvard University, 16 Divinity Ave., Cambridge, MA 02138; 3 Center for Dark Energy Biosphere Investigations, University of Southern California, 3616 Trousdale Parkway, Los Angeles, CA 90089; 4 Department of Oceanography, University of Hawai’i at Mānoa, 1000 Pope Rd., Honolulu, HI 96822; 5 Global Undersea Research Unit, University of Alaska Fairbanks, P.O. Box 475, Moss Landing, CA 95039; 6 Moss Landing Marine Laboratory, 8272 Moss Landing Road, Moss Landing, CA 95039.

## Abstract

The rock-hosted, oceanic crustal aquifer is one of the largest ecosystems on Earth, yet little is known about its indigenous microorganisms. Here we provide the first phylogenetic and functional description of an active microbial community residing in the cold oxic crustal aquifer. Using subseafloor observatories, we recovered crustal fluids and found that the geochemical composition is similar to bottom seawater, as are cell abundances. However, based on relative abundances and functional potential of key bacterial groups, the crustal fluid microbial community is heterogeneous and markedly distinct from seawater. Potential rates of autotrophy and heterotrophy in the crust exceeded those of seawater, especially at elevated temperatures (25 °C) and deeper in the crust. Together, these results reveal an active, distinct, and diverse bacterial community engaged in both heterotrophy and autotrophy in the oxygenated crustal aquifer, providing key insight into the role of microbial communities in the ubiquitous cold dark subseafloor biosphere.

Oceanic crust encompasses the largest aquifer on Earth, with a liquid volume equal to approximately 2% of the ocean’s volume^1^. It also harbors a substantial reservoir of microbial life that may influence global-scale biogeochemical cycles^2^. To date, knowledge of microbial life in basaltic crust is derived primarily from the study of crustal fluids from the warm ridge flank in the NE Pacific where anaerobic and thermophilic microbial lifestyles dominate^3^,^4^,^5^ and from the examination of basaltic rocks collected from mid-ocean spreading ridges^6^,^7^,^8^. However, very little is known about the oceanic crustal aquifer in low-temperature ridge flank hydrothermal systems, where circulating fluids are predicted to be cold (<20 °C) and oxygenated^9^. North Pond is a sedimented basin along the Mid-Atlantic ridge that is relatively well studied in terms of its geology and hydrology, and may serve as a model for thousands of similar sediment basins flanking slow-spreading mid-ocean ridges^10^. The sediment layer (<200 m thick) at North Pond provides an effective barrier to seawater-basalt exchange, while the surrounding ridges of exposed rocky outcrops facilitate the exchange of fluids between the deep ocean and the crustal aquifer. Previous work has shown that fluid flows rapidly in a horizontal direction through the porous oceanic crust beneath North Pond^11^,^12^, and oxygen profiles of the sediment column revealed that the deepest sediment contains higher concentrations of oxygen than shallower sediment, providing evidence that the basaltic aquifer is oxygenated^2^,^13^. Less well constrained are what microbial communities reside in these fluids and how this microbial life impacts biogeochemical cycling in the crustal aquifer and overlying ocean.

CORK (Circulation Obviation Retrofit Kit) subseafloor observatories, installed through scientific ocean drilling programs (e.g., Integrated Ocean Drilling Program, IODP), allow access to subseafloor fluids and enable the characterization of microbial life in low-temperature ridge-flank hydrothermal systems^14^. These CORK observatories are installed in several locations, including in the warm (64 °C), 3.5 million-year-old ridge flank of the Juan de Fuca Ridge and in the cold (<20 °C), 8 million-year-old ridge flank at North Pond. New CORK observatories in two drill holes were installed at North Pond in November 2011 during IODP Expedition 336^15^,^16^ and the first crustal fluids for microbiological analysis were collected from these boreholes six months later using a remotely operated vehicle (ROV). These CORK observatories are designed and constructed to minimize contamination and serve studies that utilize basaltic crustal fluids, allowing for samplers to be deployed within the seafloor at multiple isolated depth intervals as well as at the CORK wellhead^15^,^16^. Specifically, the CORK observatory in U1382A has a packer seal in the bottom of the casing and monitors/samples a single zone in uppermost oceanic crust extending from 90 to 210 mbsf (meters below seafloor). Hole U1383C was equipped with a three-level CORK observatory that spans a zone of thin basalt flows with intercalated limestone (~70–146 mbsf), a zone of glassy, thin basaltic flows and hyaloclastites (146–200 mbsf), and a lowermost zone (~200–331.5 mbsf) of more massive pillow flows with occasional hyaloclastites in the upper part^15^. Both CORK observatories consist of fiberglass casing within the oceanic crust^15^. Here we present the initial microbiological and geochemical characterization of the crustal fluids from cold, oxygenated igneous crust at North Pond on the western flank of the Mid Atlantic Ridge. We examined the geochemical and microbial signatures of crustal fluids to resolve the extent of geochemical transformations during passage through the crust and the presence and activities of microbes living in the circulating subseafloor fluids.

## Results

### General chemical characteristics of North Pond crustal fluids

**Figure 1:**
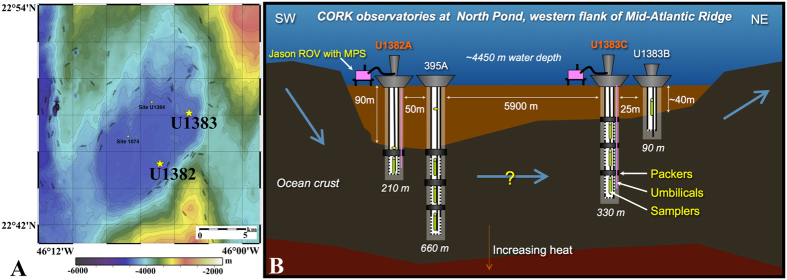
Map (**A**) and diagram (**B**) of CORK observatories at North Pond. Crustal fluids were extracted from IODP Holes U1382A and U1383C for microbial community analysis in this study. Adapted with permission from^15^.

Crustal fluids were retrieved from the single horizon at IODP Hole U1382A and from the shallow, middle, and deep horizons at IODP Hole U1383C (Fig. 1). Slightly lower levels of dissolved oxygen were detected in crustal fluids from both boreholes than in bottom seawater (Table 1). We attempted to measure hydrogen sulfide and iron species at the well head using *in situ* voltammetry, as well as on deck using a shipboard voltammetic analyzer, but they were not detected (detection limit 0.1 μM for HS- and 1 μM for Fe(II)). Hydrogen sulfide, hydrogen, and methane were also below the limits of detection by the shipboard mass spectrometer on deck (limits of detection ~500 nM for H_2_, ~500 nM for CH_4_, ~1 nM H_2_S). Concentrations of the dissolved elements Si, V, Cs, Ba, and U differed between crustal fluids and bottom seawater, with crustal fluids having more V, Si, Cs, Ba but less U than bottom seawater (Table 2). In contrast, concentrations of dissolved Sr, B, Mo, Mn, Fe, Li, Rb, S (Sulfate), Na, Ca, Mg, K, N_2_, CO_2_, and pH were indistinguishable within analytical uncertainties between crustal fluids and seawater (Tables 1 and 2). Dissolved organic carbon (DOC) concentrations in basement fluids were lower than those in background seawater, with the highest values in borehole U1382A and the lowest values in borehole 1383C. Total dissolved nitrogen (TDN) was similar between seawater and borehole 1383C, with elevated values in borehole U1382A (Table 1). A high heterogeneity of particles was detected on the GFF filters and 3 to 9 L of crustal fluids could be filtered onto a single membrane before clogging. Particulate organic carbon (POC) ranged from 0.4 to 1.8 μmol L^−1^ in crustal fluids, relatively more abundant than POC in bottom seawater (0.1 to 0.3 μmol L^−1^, Table 3). Particulate nitrogen (PN) content was 0.01 to 0.03 μmol L^−1^ in crustal fluids, much lower than PN in bottom seawater (0.2 to 0.5 μmol L^−1^, Table 3). As a result, the POC/TN molar ratios of the particulate organic matter from basement fluids ranged from 27 to 62, while those of bottom seawater were 7 to 8.

### Cell densities

All fluid samples, including crustal fluids and bottom seawater, ranged from 1.4 to 2.2 × 10^4^ cells per ml (Table 4). Quantitative PCR detected 260 to 7500 copies of bacterial 16S rRNA genes per ng extracted DNA (Table 4). Archaeal 16S rRNA genes could not be consistently amplified and are not included in the results. Approximately 97% of cells in U1383C hybridized with the bacterial-specific FISH probe EUB338, while slightly lower proportions of cells hybridized with EUB338 in fluids from U1382A and bottom seawater (93% and 91%, respectively, Table 4). Cells hybridizing with the archaea-specific FISH probe were only observed very rarely on the filters (i.e. once every other counting grid).

### Incubations with ^13^C-labeled carbon sources

**Figure 2:**
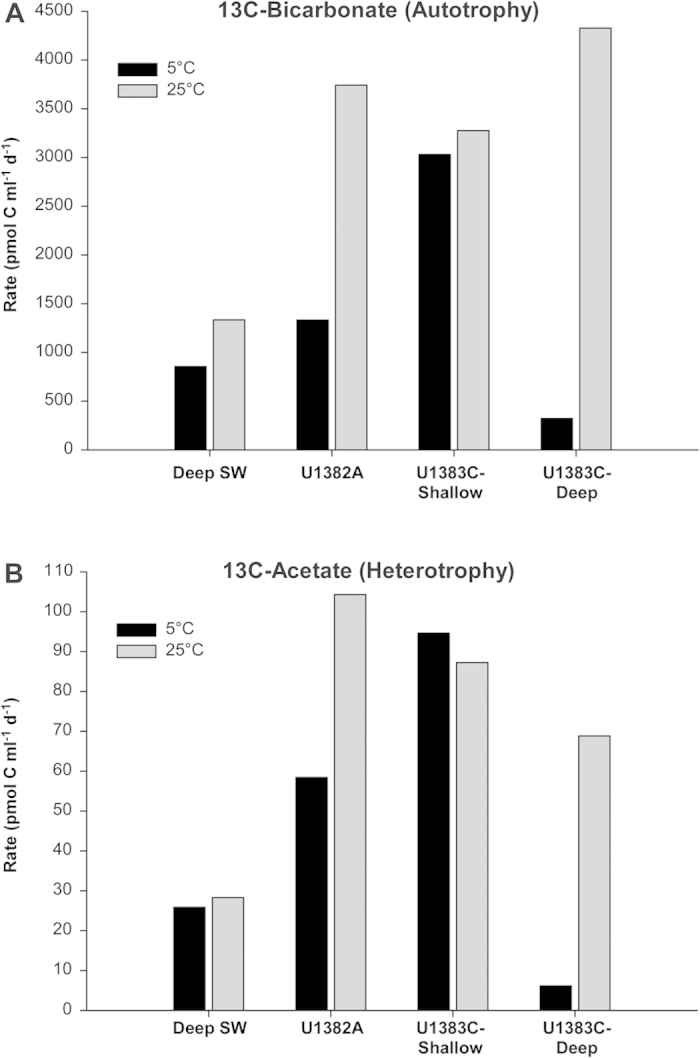
Rates of carbon transformation, including autotrophic (**A**) and heterotrophic (**B**) growth, calculated from stable isotope incubation experiments. Incubations with stable isotopes were conducted at both 5 °C (black bars) and 25 °C (grey bars). Incubated fluids include: deep Atlantic bottom seawater (SW) and crustal fluids retrieved from IODP Holes U1382A and U1383C.

Incubation of crustal fluids and bottom seawater with labeled carbon sources demonstrated the presence of cells capable of utilizing the isotopically labeled carbon sources through both autotrophic and heterotrophic activity at 5 or 25 °C (Fig. 2; Supplementary Table 1A,B). Potential autotrophic activity, as determined by the incorporation of labeled bicarbonate at atmospheric pressure, ranged from 320 to 4100 pmol C ml^−1^ day^−1^ (Fig. 2A). Potential heterotrophic activity, determined by the incorporation of labeled acetate at atmospheric pressure, ranged from 6 to 104 pmol C per ml per day (Fig. 2B). Overall, both autotrophic and heterotrophic incorporation of ^13^C was higher in the incubated crustal fluids than in bottom seawater. In most cases, the rates of autotrophy and heterotrophy were higher at 25 °C than at 5 °C. This trend was most pronounced in samples from the deepest horizon of U1383C, where very little activity was detected at 5 °C. The two shallower crustal fluids had higher levels of both autotrophic and heterotrophic growth when incubated at 5 °C than the deeper crustal fluids from U1383C at 5 °C.

### Microbial community composition via Illumina 16S rRNA and 16S rRNA gene sequencing

**Figure 3:**
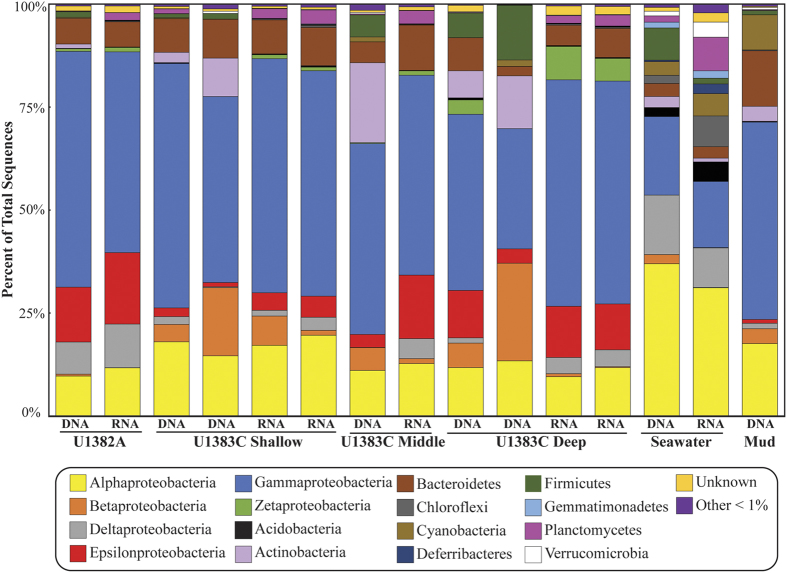
Bacterial community structure in North Pond crustal fluids based on 16S rRNA and 16S rRNA gene V6 amplicon data. Relative abundance of amplicon sequences assigned to bacterial Phyla (Class level for *Proteobacteria*) in crustal fluids from IODP Holes U1382A and U1383C, deep Atlantic bottom seawater, and drilling mud.

Genomic DNA and total RNA were successfully extracted and bacterial 16S rRNA and 16S rRNA genes were amplified from all crustal fluid samples and from bottom seawater. Approximately 72 ng of DNA was recovered for each fluid sample. Only genomic DNA was extracted from the drilling mud that was used during drilling operations to help wash cuttings from the borehole^17^, as RNA was not preserved at the time of collection. Archaeal 16S rRNA genes were not amplifiable from genomic DNA extractions. A total of 14,656,528 quality-filtered sequences of the hypervariable v6 region were obtained for bacterial amplicon libraries. At the 3% clustering level, a total of 33,287 OTUs were detected. All samples, including crustal fluids, bottom seawater, and drilling mud, were dominated by Proteobacteria, which constituted as much as 90% of reads (Fig. 3). *Gammaproteobacteria* made up 30 to 57% of the total reads and *Epsilonproteobacteria* were common only in crustal fluids (Fig. 3). The Orders *Alteromonadales* and *Chromatiales* were the most commonly detected *Gammaproteobacteria*, while most of the *Epsilonproteobacteria* belonged to the *Campylobacterales* (Supplementary Fig. 1)*. Alphaproteobacteria* and *Deltaproteobacteria* were detected in higher relative abundances in seawater than in crustal fluids. Overall, more than 80% of taxa in either the DNA or RNA fraction of crustal fluid samples were also detected in bottom seawater. While there was considerable overlap in the identity of taxa in the crustal fluids and in the bottom seawater, the relative abundances of different groups reveal a distinct crustal fluid bacterial community structure (Supplementary Fig. 2).

**Figure 4:**
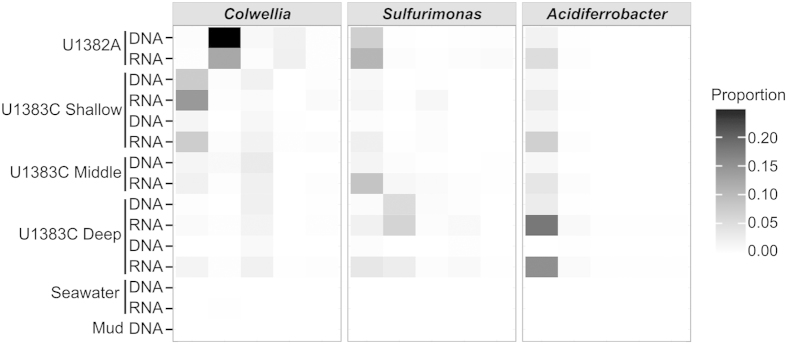
Abundant taxa in North Pond crustal fluids based on 16S rRNA and 16S rRNA gene V6 amplicon data. Proportion of sequencing reads assigned to the five most abundant OTUs (Operational Taxonomic Units) of *Colwellia*, *Sulfurimonas*, and *Acidiferrobacter* in crustal fluids from IODP Holes U1382A and U1383C, in comparison to bottom seawater and drilling mud. Each column in the heatmap represents a unique OTU.

The three most abundant genera in all crustal fluid samples were *Colwellia*, *Sulfurimonas*, and *Acidiferrobacter* (Supplementary Fig. 3). These three genera were also detected in bottom seawater or in the drilling mud at very low levels. A total of 1,502 OTUs were assigned to the genus *Colwellia*, and the dominant *Colwellia* OTUs were different between boreholes U1382A and U1383C (Fig. 4). No OTUs in bottom seawater or drilling mud DNA fractions were assigned to *Colwellia*. A total of 1,143 OTUs were assigned to the genus *Sulfurimonas*, again with the most abundant OTU varying between holes and depth horizons (Fig. 4). Less than 1% of the reads in bottom seawater and drilling mud were assigned to *Sulfurimonas*. A total of 477 OTUs were assigned to the genus *Acidiferrobacter*. All borehole samples were dominated by a single *Acidiferrobacter* OTU (Fig. 4). No OTUs in bottom seawater or drilling mud DNA fractions were assigned to *Acidiferrobacter*. In all samples, reads assigned to *Acidiferrobacter* were in higher abundance in the RNA fraction than in the DNA fraction. In contrast, the most abundant group in the DNA fraction of bottom seawater was the *Deltaproteobacteria* clade SAR324 (Fig. 3). *Chloroflexi* clade SAR202 was the most abundant group in the RNA fraction of bottom seawater. The most abundant group in the drilling mud, which is prepared with surface seawater, was *Alteromonas*.

While many taxa occurred at relatively low abundances in the DNA and RNA fractions (Supplementary Table 2), the dominant genera, *Colwellia*, *Sulfurimonas*, and *Acidiferrobacter*, were usually detected in higher abundances in the RNA fraction. Overall, the community composition of the DNA fraction was significantly different from the RNA fraction in crustal fluids (ANOSIM statistic *R* = 0.68, p = 0.003) and OTUs assigned to *Colwellia*, *Sulfurimonas*, and *Acidiferrobacter* contributed at least 12% of this difference. Other groups that contributed most to the difference between RNA and DNA fractions included *Mariprofundus*, JTB255 Marine Benthic Group, *Alteromondaceae* C1-B045, and *Marinobacter*, all of which were generally detected at higher abundances in the RNA fraction.

### Metagenomic results

Details of assembly statistics and annotation results can be found in the Supplementary Online Material (SOM). 671 putative full-length 16S rRNA genes were assembled from the identified 16S rRNA fragments, with 167 ± 75 putative genes per sample. Based on the length-normalized abundance estimates determined by EMIRGE, many of the putative 16S rRNA genes represent organisms with <1% abundance (8.2–64.9% of putative genes) (Supplementary Fig. 4). For the bottom water sample, most of the groups >1% abundance belonged to the *Alphaproteobacteria*, while U1382A possessed a nearly equivalent abundance of *Epsilonproteobacteria* (25%) and *Gammaproteobacteria* (22%). The U1383C deep sample was almost entirely assigned to the *Epsilonproteobacteria* (84%), while the U1383C shallow sample had more sequences assigned to the *Gammaproteobacteria* compared to the *Epsilonproteobacteria* (Supplementary Fig. 4).

**Figure 5:**
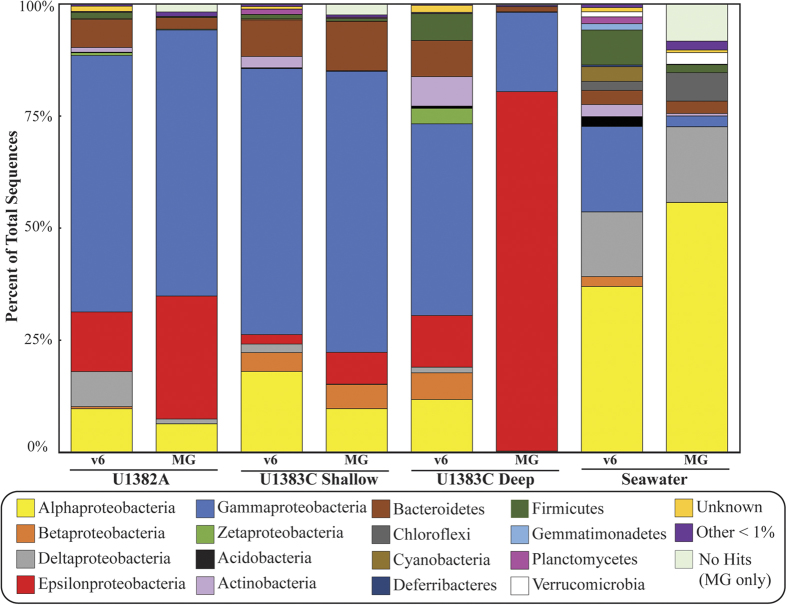
Comparison of relative abundance and taxonomic assignment at the Class level for bacteria in North Pond crustal fluids and seawater from 16S rRNA gene V6 amplicon data and metagenomic libraries based on essential marker genes.

Using the essential marker genes described in Albertsen *et al*.^18^, 12,774 putative marker genes were identified from the North Pond samples. These putative markers genes were assigned taxonomies at the Class level, if available, and used to recruit sequences from size-normalized metagenomic libraries. In general, the number of putative markers without an assignment at the Class level was small (12–36%), as was the percentage of markers assigned to groups with <1% abundance (2–5%). The metagenome sequences of the bottom water predominantly represented groups within the *Alphaproteobacteria*, while in U1382A, *Epsilonproteobacteria* and *Gammaproteobacteria* were represented in nearly equivalent proportions (Fig. 5). The deep U1383C sample recruited almost exclusively sequences to *Epsilonproteobacteria* markers (80%), with a few other minor bacterial groups. The shallow U1383C sample had a larger proportion of *Gammaproteobacteria* sequences (41%) than any other taxonomic group (Fig. 5). The taxonomic assignment based on essential marker genes was similar to the taxonomic assignments from amplicon sequencing of the 16S rRNA gene v6 region, with the exception of U1383C Deep, where the metagenomic profile indicates ~75% *Epsilonproteobacteria*, while amplicon sequencing suggests they are present at ~15% (Fig. 5).

**Figure 6:**
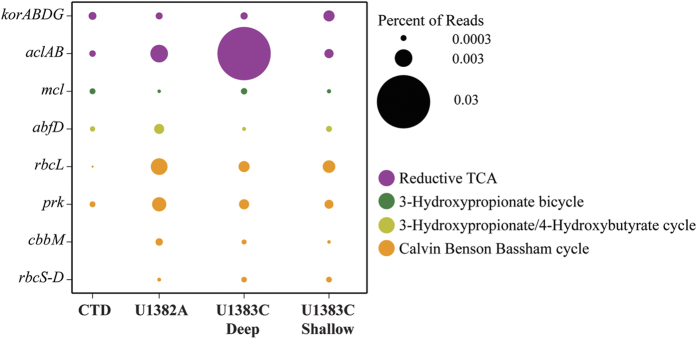
Carbon fixation pathways in North Pond crustal fluid metagenomes. Percent of reads annotated to genes for 4 carbon fixation pathways for the 4 metagenomes from IODP Holes U1382A, U1383C, and bottom seawater. kor, 2-oxoglutarate ferredoxin oxidoreductase; acl, ATP-citrate lyase; mcl, malyl-CoA lyase; abfD, 4-hydroxybutyryl-CoA dehydratase; rbc, ribulose-bisphosphate carboxylase; prk, phosphoribulokinase; rbcL and cbbM, ribulose-1,5-bisphosphate carboxylase/oxygenase.

Putative coding DNA sequences (CDS) from the North Pond IMG annotations were matched to various essential genes for the dominant known carbon fixation pathways. Sequences were recruited from size-normalized metagenomes to each putative carbon fixation gene to determine which forms of carbon fixation potentially dominate in each sample from the aquifer (Fig. 6). There was no indication of the reductive acetyl-CoA pathway in any of the North Pond samples. Genes related to the 3-hydroxypropionate bicycle and the 3-hydroxypropionate/4-hydrocybutyrate cycle were detected in some of the samples, but with very low representation in the libraries. However, in the crustal fluid samples, both reductive Citric acid (rTCA) and Calvin-Benson-Bassham (CBB) cycles were relatively abundant, with the rTCA cycle being most highly represented in the U1383C deep horizon (Fig. 6). Conversely, in the shallow horizon at U1383C, the genes associated with the CBB cycle were the more abundant, with two forms of RuBisCo and only minor contributions from ATP-citrate lyase (*aclAB*) (Fig. 6). Assignment of putative taxonomies to the putative aclAB genes demonstrates that for the crustal fluids, the predominant organisms associated with the rTCA cycle belong to the *Epsilonproteobacteria*, which were not identified in the bottom seawater sample (Supplementary Fig. 5). In some cases, taxonomic assignments of the putative CDS could be assigned to the Order *Campylobacterales* and Family *Helicobacteraceae*, where the *Sulfurimonas* lineage resides (Supplementary Fig. 5).

Using the fraction of putative CDS assigned to TIGRFAMs as a signature for potential large-scale functional differences for each North Pond and several publicly available metagenomes, a principal component analysis was performed to visualize how the various metagenomes relate to each other (Supplementary Fig. 6). The crustal fluid North Pond samples cluster together away from the corresponding bottom water sample. Further, metagenomes representing oxic, surface-influenced sediment (south Pacific abyssal sediment, 5 cmbsf), anoxic, deep sediment (Arctic ridge, 75 cmbsf) and metal-rich hydrothermal plume waters (Guaymas and Abe Lau), were all distinct from the North Pond crustal fluid samples (Supplementary Fig. 6).

## Discussion

The geochemical attributes of these fluids suggests a high degree of connectivity between the cold crustal aquifer at North Pond and deep Atlantic seawater. Many of the dissolved major and minor elements and other geochemical tracers in crustal fluids were indistinguishable in concentration, and in some cases isotopic composition, from seawater. It should also be noted that both of these boreholes were drilled ~6 months prior to sampling, a procedure that involves injecting surface seawater into the hole along with periodic flushes of drilling mud. Additionally, both holes were located within 50 m of open boreholes that penetrated into the basement and continued to accept an influx of bottom seawater until they were sealed in 1997 and 2012^11^,^19^,^20^. Each of these factors may impact the local hydrology and geochemistry in the crustal aquifer, but the extent of that impact is difficult to constrain. Nevertheless, the data herein do reveal that the fluids sampled are, at the very least, partly if not mostly derived from the formation. For example, there are indicators that crustal fluids are reacting with rocks and/or sediments within the aquifer, resulting in dissolved concentrations of elements that are distinct from background seawater, such as the lower uranium concentrations in U1383C crustal fluids than in seawater, that is consistent with the removal of U into minerals during water-rock reactions^21^. The higher concentrations of dissolved silica in the crustal fluids may result from either diffusive exchange with sediment pore waters or water-rock reactions^22^. Sediment contains opal, which has the highest solubility of all Si-bearing minerals in the crust, and has been shown to impact borehole conditions^22^,^23^. Alternatively, water-rock reactions at the low temperatures within these boreholes could also produce dissolved silica without a measurable calcium or magnesium anomaly^24^. Similarly, the distinct increases in the concentrations of vanadium, cesium, and barium are likely due to diffusive exchange with sediment pore waters^22^. Finally, oxygen concentrations were lower than background seawater in all horizons at U1383C, but higher than in the basal sediments^2^,^13^. This suggests that abiotic and/or biotic consumption of oxygen occurs within the aquifer, either from the oxidation of reduced minerals and microbial oxygen consumption in basalt or from diffusive losses to the overlying sediment pore waters where it is likely consumed by microbes as described in Ziebis *et al*.^13^. The DOC concentrations in the North Pond crustal fluids are consistent with those previously reported for the crustal fluids from eastern flank of the Juan de Fuca Ridge, demonstrating that ridge flank aquifer is a sink for DOC in the deep ocean^25^,^26^. The magnitude of decrease in DOC concentrations in U1382A and U1383C (all three horizons) crustal fluids relative to bottom seawater DOC concentrations is very similar to the amount of loss of oxygen from bottom seawater (~7 and ~25 μM), suggesting that oxidation of DOC may be a possible explanation to the loss of oxygen. POC and POC/TN ratio were higher in crustal fluids relative to bottom seawater, suggesting differences in particulate organic carbon and nitrogen cycling in the subseafloor compared to the deep ocean, perhaps reflecting the consumption of readily available carbon and nitrogen sources and the accumulation of recalcitrant particulate organic carbon in the subseafloor^27^,^28^,^29^.

Stable isotopic experiments demonstrate that some microbes resident in the North Pond fluids are poised to perform both autotrophic and heterotrophic growth when provided with inorganic carbon and a simple organic acid as carbon sources. This is in contrast to recent stable isotopic experiments with subsurface basalts from the same North Pond boreholes (U1382A, U1383C) that found carbon fixation rates were below detection^30^. The contrasting results may reflect the different incubation conditions used, as our work used more bicarbonate, with a lower fraction of labeled carbon, or it may reflect the loss of biomass from the basalts during the decontamination process. Previous studies of acetate respiration in sediment cores from North Pond showed generally low rates in most samples (<200 pmol respired acetate cm^−3^ d^−1^), but spikes of higher respiration (up to 10,600 pmol cm^−3^ d^−1^) at different depths that corresponded to a change in the lithology^31^. In addition, the authors detected no lag time in the utilization of acetate, indicating that some North Pond sediment microbes, while existing in an oligotrophic habitat, are poised to take advantage of labile carbon sources as they become available. Similarly, we measured generally low potential rates (up to 104 pmol respired acetate cm^−3^ d^−1^) of acetate respiration in crustal fluids. While rates of potential heterotrophy in shallow crustal fluids exceeded that of deep crustal fluids and bottom seawater, potential autotrophy in the deepest horizon at 25 °C exceeded all others. These results suggest differences in the functional capacity of microbial communities in the different depth horizons and that autotrophy may be occurring in this community. This may be due to limitations in organic carbon abundance and quality, or other factors that have yet to be determined. Data from previously deployed thermistor arrays at North Pond suggests basement temperatures up to 20 °C at approximately 600 mbsf^11^, and we saw that the potential rate of carbon utilization increased with elevated temperatures, which likely reflects increased reaction kinetics or the increased activity of microbes that are adapted to warmer temperatures, such as those found with increasing depth in the crust.

The 16S rRNA and 16S rRNA gene amplicon sequencing from crustal fluids, seawater, and drilling mud, together with the metagenomic analysis of select samples, revealed a diverse microbial community in the North Pond crustal fluids that is distinct from deep seawater communities. The crustal fluids are dominated by *Alpha*- *Epsilon*-, and *Gammaproteobacteria* unlike the population structure seen in sediments, deep seawater, or hydrothermal habitats (as reviewed in^32^). Amplicon sequencing identified some key subseafloor organisms in the aquifer, including one potential group of iron-oxidizing bacteria, *Acidiferrobacter*. While there is only one named isolate of *Acidiferrobacter* (an iron- and sulfur-oxidizing diazotroph formerly known as *Acidothiobacillus*)^33^, this genus has been detected in many different habitats in the deep sea including sediment, basalt, hydrothermal habitats, and deep seawater^32^. Although iron levels in crustal fluids were below the detection limit, microbes in the subseafloor likely access iron through attachment to basalt rocks. Other putative iron oxidizers, including *Mariprofundus* and *Marinobacter*, were also detected in the 16S rRNA amplicon libraries at higher abundance than in the DNA fractions, suggesting iron-based energy metabolism may be important in the crustal aquifer. *Colwellia* was the most abundant *Gammaproteobacteria* detected. This group is among the common denizens of the pelagic ocean^32^ and cultivated isolates of *Colwellia* include obligate piezophiles and psychrophiles^34^,^35^,^36^,^37^,^38^. *Colwellia* has previously been isolated through aerobic enrichment cultures from basalt, but were not found exclusively in basalt-hosted environments^35^. Despite the ubiquity of *Colwellia* in deep seawater, the high relative abundance of this group in crustal fluids rather than in bottom seawater at North Pond suggests that it may be favored by conditions found in the aquifer. Finally, the presence and high relative abundance of *Sulfurimonas*, particularly in the deepest North Pond crustal fluids, is especially striking. *Sulfurimonas* is a widespread resident of deep-sea habitats, including marine sediment, oxygen minimum zones, hydrothermal vents and plumes, ridge flanks, and inactive sulfide chimneys^39^,^40^,^41^. *Sulfurimonas* was also detected in sediment from North Pond collected during IODP Exp 336 in 2009 (B. Orcutt, personal communication). This interaction between sediment and the crustal aquifer is also evident in the presence of oxygen in the sediment at the sediment-basement interface at North Pond^2^, which would support microbes like *Sulfurimonas* that preferentially straddle gradients between oxic and anoxic habitats. However, in the deepest horizon of U1383C, there are different *Sulfurimonas* OTUs than those seen in the shallow horizon of U1383C or at U1382A, and the metagenomic analysis showed that the deepest horizon is dominated by *Sulfurimonas*. This suggests that at depth in the crust, far away from the overlying sediment, fluids and their microbial communities may be isolated, perhaps due to reduced permeability of oceanic crust as cracks are filled with clays and alteration minerals, thus influencing the biogeochemical state and microbial communities within crustal fluids^42^.

Furthermore, clear differences in the relative abundance of dominant genera in the DNA and RNA fractions of North Pond crustal fluids were detected using amplicon sequencing of the bacterial 16S rRNA and 16S rRNA gene. The most dominant groups in the RNA fraction may reflect the groups that are most active in these crustal fluids, or may reflect groups with more ribosomes per cell. In either case, the most abundant genera in the RNA fraction likely have the highest potential for protein synthesis. While any functional assignments based on taxonomy are purely speculative, it is possible that North Pond *Colwellia* are heterotrophic and contribute to the observed transformation of acetate. Likewise, both the *Sulfurimonas* and *Acidiferrobacter* that dominate the RNA fraction of North Pond crustal fluids have the potential to be autotrophic and contribute to the carbon fixation documented by the stable isotopic experiments. The metagenomic analysis of carbon fixation genes provides further support for a community capable of autotrophy in the aquifer, particularly among the *Sulfurimonas* deep in the aquifer.

The differences in the microbial community structure between North Pond crustal fluids and bottom seawater provide several significant insights into the ecology of the basalt-hosted biosphere. First, although there are similar cell densities in warm, reducing, and highly altered (64 °C) crustal fluids collected from the Juan de Fuca ridge flank^5^ and cold, oxic, and unaltered crustal fluids at North Pond, the two sites have vastly different microbial communities. This is not surprising given the substantial differences in the chemical characteristics of these fluids. The data herein also reveal that the microbial communities in the North Pond aquifer are notably different in both taxonomic and functional composition than those in the deep Atlantic bottom water. However, this is surprising in light of the thermal and chemical similarities of the aquifer fluid and the bottom water. This suggests that the crustal structure (e.g., permeability, hydrogeology, effective porosity) and/or composition (e.g., mineralogy, bulk composition) may be important factors in shaping this subsurface microbial community. In addition, our results suggest that autotrophy occurs in the North Pond crustal fluids, likely supported by the availability of electron donors and acceptors such as iron and sulfur in the basaltic crust. This could also alleviate competition for labile organic carbon, which is likely to be sparse in these deep oligotrophic waters. Future metagenomics analysis will focus on determining the potential energy acquisition pathways for the dominant organisms and what metabolisms drive these communities. Together, the observed differences in potential rates of both heterotrophic and autotrophic carbon utilization between the horizons in U1383C, as well as the differences in the distribution of strains and carbon fixation genes of the dominant taxa (especially *Sulfurimonas*), provide compelling support for the hypothesis that crustal fluids may be more isolated with depth at North Pond. Genomic reconstruction of key subseafloor lineages will help assess the evolutionary relationship, functional capacity, and potentially the physical connection, if any, of microbes from different depths beneath the seafloor.

These first results from the oxygenated crustal aquifer contribute to our broader understanding of the deep subseafloor, as well as carbon processing in the cold, dark biosphere. We know that the vast flow of fluid exchanging between ocean basins and crustal reservoirs plays an important role in mediating transport of heat and solutes in the oceans, and this study provides the first insight into microbial life and its potential impact on biogeochemical cycles in the global cold, oxygenated crustal aquifer.

## Materials and Methods

### Sample collection

Crustal fluids were collected from the single horizon at U1382A and from the shallow, middle, and deep horizons at U1383C^15^ using an ROV-based pumping and filtration system tailored for microbial sampling. The mobile pumping system (or MPS)^43^ consists of a plumbing flow path that is primed with sterile DI water prior to deployment and includes sensors for measuring real time flow rate (Seametrics), temperature (Seabird), dissolved oxygen and temperature (Aanderaa), and redox analytes (O_2_, HS-/H_2_S, Fe(II), using *in situ* voltammetry). The MPS was mounted on the ROV *Jason II* and attached to one of the connectors on the wellhead that emerges via an umbilical to the hydrologic horizon of interest within the crustal aquifer. The MPS was used to pump fluids up a 1.25 cm inner diameter Tefzel tubing that is part of the CORK’s umbilical system. Individual CORK fluid delivery lines were flushed using the MPS at a rate of ~4 liters per minute for at least 3 times volume of the fluid delivery line (~20–30 minutes) prior to diverting fluid flow to six 15 L foil-lined tedlar™ sample bags (Jensen Inert Products) that were acid cleaned and sterilized using gamma irradiation prior to deployment. Oxygen concentrations and temperatures were measured every minute during flushing, and fluids were not sampled until at least 15–30 minutes of stable, reproducible measurements were observed, indicating a fully flushed fluid delivery system and access to pristine crustal fluids. In addition, ~5 L of fluid from each of the three horizons in U1383C was filtered *in situ* onto 47 mm SUPOR filters in pancake-style filter holders (McLane Inc). These filtered samples were preserved *in situ* using a reservoir of RNA Later (Qiagen) that is part of the MPS pumping system^43^. Organic particles for carbon and nitrogen analysis were filtered *in situ* onto 25 mm glass microfiber filter (GFF). Once recovered, ten liters of each sample was filtered onto a 0.22 μm Sterivex-GP filter at 5 °C for microbial analysis. Similarly, bottom seawater was collected by CTD at 100 m above the sea floor and filtered in the same manner. *In situ* and ship-based filters were fixed at 4 °C for 18 hours with RNA Later immediately after filtering or upon recovery, then frozen at −80 °C until nucleic acid extractions. A frozen sample of drilling mud was obtained from U1382A during IODP Exp. 336^16^.

Aliquots of recovered fluids were placed in acid-washed 1000 mL high-density polyethylene (HDPE) bottles, 500 and 1000 mL glass bottles, and 10 to 50 mL glass ampoules for shore-based geochemical analyses. Some of these aliquots were acidified with trace metal clean 6N HCl to a final pH of 1.8. Other aliquots were frozen for nutrient analysis and preserved with a dilution of 1:200 with saturated HgCl_2_.

### Chemistry methods

Concentrations of the major ions in seawater (Na, Mg, Ca, S [sulfate], K, and Sr) were determined using an Inductively Coupled Plasma-Optical Emission Spectrometer (ICP-OES) and by diluting samples 1:200 with ultra-pure water (yielding a precision of 1% at the bottom seawater value). Concentrations of B, Mn, Fe, Si, and Li were also measured with an ICP-OES diluted to 1:25 to yield a precision of ~1 to 2% at the bottom seawater value, with the exception of Mn and Fe which have bottom seawater concentrations that are below detection (<0.1 μmol kg^−1^). At a concentration of 4.5 μmol kg^−1^ the precision is 2%. An identical precision is determined for Fe measurements at concentrations of 4.4 μmol kg^−1^. Concentrations of the trace ions V, Ba, Mo, Rb, Cs, and U were determined using an inductively coupled plasma - mass spectrometer (ICP-MS) with a dilution of 1:75 and an analytical precision of 3% at the bottom seawater value. Concentrations of Si, nitrate, ammonium, and phosphate also were determined colorimetrically^44^ with a precision of about 2% . Chlorinity was determined by potentiometric titration with AgNO_3_ with a precision of 0.2% of the seawater concentration.

Dissolved volatiles (H_2_, H_2_S, CH_4_ and CO_2_) were determined shipboard by analyzing 4 L of fluid directly from unopened, unaltered tedlar™ gastight bags (or deep water samples collected via CTD casts) using a Stanford Research Systems Inc. quadropole mass spectrometer equipped with a custom-membrane inlet and a peristaltic pump and Norprene™ tubing^45^. Values were estimated by analyzing representative discrete spectra of deep bottom water and borehole water, and using existing calibration curves to determine the relative differences among samples. Bottom seawater concentrations are derived from existing literature. Volatile concentrations shown are ±10%. On deck, discrete samples were also analyzed using voltammetry with an inline flow cell fluid path for comparison *in situ* voltammetry. pH was determined in triplicate using a double junction electrode and a pH meter (Radiometer Inc).

Dissolved organic carbon (DOC) and total dissolved nitrogen (TDN) were measured by high-temperature (680 °C) combustion using a Shimadzu TOC-L analyzer at the SOEST Laboratory for Analytical Biogeochemistry, University of Hawaii. Samples analyzed include those filtered *in situ* for crustal fluids as well as the CTD samples filtered on deck. Samples were acidified to pH < 2 within the autosampler syringe and were purged with nitrogen to remove inorganic carbon. Five to six replicate analyses were performed using an injected sample volume of 150 μL. The detection limits of DOC and TDN were about 2 and 1.5 μM, respectively. Two consensus reference materials CRM, from University of Miami^46^, deep seawater and low carbon water, were used extensively before, between, and after sample analysis to monitor analytical accuracy. The analytical reproducibility for DOC and TDN is better than 1.1 μM and 0.2 μM, respectively, by repeated analysis of four deep seawater CRM. Carbon and nitrogen concentrations of particulate organic matter were measured by the Biogeochemical Stable Isotope Facility at School of Ocean and Earth Science and Technology, University of Hawaii. A few drops of sulfurous acid (6–9% of H_2_SO_3_) were added to wet the filter and to remove inorganic carbon from the filter. The acidified filters were transferred to a 60 °C oven for 24 hours. The dried filters were then transferred into a tin capsule before placing within a high-temperature combustion CN elemental analyzer (Costech, ECS 4010) connected in-line with a Mass Spectrometer (ThermoFinnigan Delta XP interfaced with a ConFloIV) for analysis of their nitrogen isotopic compositions. The detection limit for carbon and nitrogen content is 10 μg-C and 0.3 μg-N, respectively. The limit for reliable nitrogen isotopic determination is 10 μg-C and 10 μg-N, respectively.

### Microbial cell counts with DAPI and CARD-FISH

Whole crustal fluids and bottom seawater were fixed with 3.7% formaldehyde for cell counts. Up to 19.8 ml of fixed fluids were filtered onto a 0.2 μm GTBP polycarbonate filter (Millipore Inc), stained with DAPI (4′,6′-diamidino-2-phenylindole; Sigma), and counted via epiflourescent microscopy^47^. For catalysed reported deposition fluorescence *in situ* hybridization (CARD-FISH), cells were filtered onto 0.2 μm GTTP polycarbonate filters (Millipore Inc) and fixed with 2% paraformaldehyde, rinsed with milliQ H_2_O, air dried and stored at −20 °C until further use. Cells on filters were hybridized with HRP-labeled 16S rRNA targeted oligonucleotide probes EUB338^48^, ARCH915^49^ and NON338^48^ (Biomers GmbH, Ulm, Germany), and the signal was amplified as described elsewhere^50^ using Alexa 488^®^ tyramides (Invitrogen). The permeabilization step of the protocol before probe hybridization was modified, such that the cells on the filters were first permeabilized with Proteinase K (0.005 U μl ^−1^ in 0.05 M EDTA, 0.1 M Tris-HCl, at pH 8) for 30 minutes at 37 °C. Filters were then washed in 50 ml 1X PBS at room temperature, followed by a second permeabilization treatment with Lysozyme (10^6^ U ml^−1^, in 0.05 M EDTA, 0.1 M Tris-HCl, at pH 8) for 30 minutes at 37 °C. After signal amplification, all cells were counterstained with DAPI and counted via epiflourescent microscopy.

### Quantitative PCR (qPCR)

The relative abundance of bacterial and archaeal 16S rRNA genes was determined by qPCR assays as previously described^51^,^52^. To generate standards, plasmid DNA was extracted from Axial Seamount low-temperature diffuse vent clone libraries, purified, and linearized using the WizardPlus SV Minipreps DNA Purification System (Promega Inc). Standards were constructed by mixing equal amounts of four bacterial plasmids for the quantification of bacterial 16S rRNA gene and two archaeal plasmids for the quantification of archaeal 16S rRNA gene. A 1:10 dilution series of the plasmid mixtures beginning with an initial concentration of 0.06 ng μl^−1^ (bacteria) and 0.10 ng μl^−1^ (archaea) was used to produce standard curves with *R*^*2*^ values >0.991 and with efficiency ranging from 93 to 96%. Each 20 μl reaction contained KAPA PROBE FAST ABI Prism^®^ 2X qPCR Master Mix (Kapa Biosystems Inc), forward and reverse primers at optimized concentrations of either 3 nM (bacteria) or 4 nM (archaea), optimized probe concentrations of either 2.5 nM (bacteria) or 5.0 nM (archaea), DEPC-treated water, and 2 μl of DNA template. Triplicate reactions were performed on a StepOne Plus Real Time PCR System (Applied Biosystems Inc) for each sample and for no template controls. Cycles began with initial denaturation for 3 min at 96 °C, followed by 40 cycles of 15 s at 96 °C and 3 min at 59 °C. STEPONE software version 2.2.2 (Applied Biosystems Inc) was used to analyze the results.

### Incubations with ^13^C-labeled carbon sources

To determine potential rates of autotrophic and heterotrophic metabolism within crustal aquifer fluids and deep Atlantic bottom water, fluids were incubated with either ^13^C-labeled bicarbonate (autotrophy) or ^13^C-labeled acetate (heterotrophy) at atmospheric pressure. Sterile, butyl stoppered 25-ml Balch tubes were prepared under N_2_ gas in an anaerobic chamber with resazurin (20 μM final concentration) with a mix of either unlabeled bicarbonate and 10% ^13^C-labeled bicarbonate (to a final concentration of 1.8 mM NaHCO_3_ and 0.2 mM NaH^13^CO_3_) or unlabeled acetate and 10% ^13^C-labeled acetate (to a final concentration of 13.5 μM C_2_H_3_NaO_2_ and 1.5 μM ^13^C_2_H_3_NaO_2_). At sea, the pre-amended Balch tubes were filled with 20 mL of freshly sampled fluids using a sterile syringe, with overpressure released by insertion of a second hypodermic needle. Incubated tubes were monitored for color changes and incubations were stopped when an observable color change in the resazurin indicated a change in the redox potential inside the tube (ie. consumption of oxygen). Sterile controls were set up as described above but with an additional filter (0.2 μm pore size) inserted between the syringe outlet and the hypodermic needle. Tubes were incubated in the dark at either 5 or 25 °C. Incubations were stopped at distinct time intervals by addition of either 0.5 mL of a 1 M NaOH solution (for incubations with bicarbonate) or 4 mL of a 20% zinc acetate solution (for incubations with acetate). Tubes were stored at −20 °C until further processing.

For analysis of ^13^C-labeled biomass, tubes were thawed and the residual pool of bicarbonate or acetate was removed by acidification to a pH of 2 by adding 25% HCl (molecular grade) while stirring and sparging with N_2_ for at least 30 minutes. The entire volume was then filtered through a pre-combusted glass fiber filter (25 mm diameter, 0.7 μm particle retention, Whatman, UK). The filters were dried in a desiccator overnight and stored at 5 °C until further processing. Filters were weighed into tin capsules and analyzed for ^13^C/^12^C ratios with an automated Isotope Cube elemental analyzer (Elementar, Germany) interfaced to a Delta Advantage isotope ratio mass spectrometer (Thermo, Germany). Rates of potential autotrophic metabolism (carbon fixation from ^13^C-labeled bicarbonate) and heterotrophic metabolism (degradation of ^13^C-labeled acetate) were calculated from δ^13^C of the theoretical carbon pool at the start and the end of the incubations (time intervals of not more than 13 days). For this, the transfer of ^13^C between pools was calculated, according to the following equation:


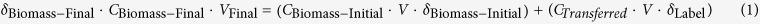


where *δ* is the isotopic ratio ((R_sample_/R_standard_ − 1) · 1000), *V* is the volume of the incubation (20 mL) and *C* is the theoretical concentration of the carbon pool based on added labeled carbon sources and assumed endogenous dissolved inorganic carbon (DIC) and dissolved organic carbon (DOC). For incubations with ^13^C-labeled bicarbonate we assumed a concentration of 2.3 mM endogenous DIC in addition to the added mix of ^13^C-labeled bicarbonate, thus decreasing the amount of label in the substrate pool. For incubations with ^13^C-labeled acetate we assumed a concentration of 45 μM endogenous DOC in addition to the added ^13^C-labeled acetate.

### Nucleic acid extraction, Illumina tag sequencing, and analysis

Sterivex filters and 47 mm flat filters were cut into two equal pieces using sterile technique. Total genomic DNA was extracted from one half using a phenol chloroform method as previously described^53^ and RNA was extracted from the other half with a *mir*Vana miRNA isolation kit (Ambion Inc) preceded by a bead beating step using RNA Powersoil beads (MoBio). Extracted RNA was treated with Turbo DNase (Ambion Turbo DNA-free kit) and converted to cDNA with an Applied Biosystems (ABI) High Capacity RNA to cDNA kit prior to amplicon library preparation. Total genomic DNA was extracted from approximately 1 g of drilling mud using a MoBio UltraClean^®^ Soil DNA Isolation Kit. The V6 region of 16S rRNA and 16S rRNA genes was amplified in triplicate for each sample with previously reported primers designed for archaea and bacteria^54^ that were modified to include indices and barcodes compatible with the Illumina HiSeq1000 platform rather than 454 Life Sciences Adapters^55^. Triplicate PCR amplifications were pooled for each sample, cleaned with a Qiagen MinElute kit, and quanitified by PicoGreen assay on a Turner Biosystems spectrophotometer. Fifty nanograms of each cleaned amplicon library was then size selected with a 2% agarose PippinPrep cassette to produce a narrow range of fragment sizes from 200 to 240 bp for sequencing and cleaned again to remove agarose. Equimolar amounts of pooled amplicon libraries and a metagenomic library were run in the same lane to avoid known difficulties of sequencing low-complexity amplicon libraries with Illumina^56^.

Paired Illumina sequencing reads were quality filtered to remove any reads containing ambiguous nucleotides and only pairs with perfectly overlapping reads were used for further analysis^55^. Raw sequencing reads are publicly available through the NCBI under BioProject PRJNA280201. Sequences were clustered at 97% similarity with a minimum word length of 30, using usearch^57^. Taxonomy was assigned by global alignment for sequence taxonomy (GAST)^58^ with the SILVA 111 database^59^. Analysis of similarities (ANOSIM) and similarity percentage (SIMPER) were performed in R using VEGAN v2.0-8^60^. Community structure was analyzed in R with phyloseq^61^ and plotted with ggplot2^62^ and phinch^63^.

### Metagenomic sequencing

DNA from the Sterivex samples collected from bottom seawater, U1382A, U1383C Shallow, and U1383C Deep was sheared to 175 bp using a Covaris S-series sonicator. Libraries were constructed using the Ovation Ultralow Library DR multiplex system (Nugen) following manufacturer’s instructions. Paired-end sequencing was performed on an Illumina HiSeq 1000 at the W.M. Keck sequencing facility at the Marine Biological Laboratory. Details of sequence trimming and assembly are described in the SOM. Raw metagenomic sequencing reads are publicly available through the NCBI under BioProject PRJNA280201.

Initial putative CDS were determined for contigs generated from each sample using Prodigal v2.6.1 (parameters: -m -p meta -q)^64^. Prodigal was used to generate putative CDS for the sediment metagenomes used for functional comparison (see below). Prodigal-predicted CDS for the NP samples were compared to publicly available deep-sea metagenomes. Additional metagenomes from deep-sea environments that also used Illumina HiSeq were accessed from IMG for comparison to the NP samples as described in the SOM. Additional comparative analyses of these metagenomes are described in the SOM. Each of the NP samples was processed through the IMG/M annotation pipeline (GOLD Analysis Project ID: Bottom water - Ga0071103; 1382A - Ga0071100; 1383C Deep - Ga0071101; 1383C Shallow - Ga0071102)^65^. IMG/M annotations were used for all searches involving metabolisms of interest and assignments of phylogenetic markers.

Full-length 16S rRNA genes were assembled and identified from each North Pond metagenome using Meta-RNA^66^ and EMIRGE^67^ as described in the SOM.

Carbon fixation genes and their taxonomic affiliation were determined in each North Pond metagenome as described in the SOM. Utilizing a method detailed previously^18^, the putative CDS from the NP sample IMG annotations were searched against a database of 100 essential phylogenetic markers using Hidden Markov models (HMMs) as described in the SOM.

## Additional Information

**How to cite this article**: Meyer, J. L. *et al*. A distinct and active bacterial community in cold oxygenated fluids circulating beneath the western flank of the Mid-Atlantic ridge. *Sci. Rep*. **6**, 22541; doi: 10.1038/srep22541 (2016).

## Supplementary Material

Supplementary Information

## Figures and Tables

**Table 1:** Dissolved oxygen, nitrogen gas and carbon dioxide, pH, and dissolved organic carbon (DOC) and total dissolved nitrogen (TDN) in North Pond subsurface crustal fluids and the overlying deep Atlantic bottom seawater.

Sample	Depth (mbsf)	Temp. (°C)	O_2_; μmol L^−1^ ± std dev (*N*)	~tCO_2_(mmol L^−1^)	pH	DOC (μM)	TDN (μM)	NO_3_^−^ (μmol L^−1^)
U1382A	90–210	3.1	244 ± 1 (*18*)	n/d	7.50 ± 0.03	43	87	21.1
U1383C Shallow	70–146	3.7	216 ± 1 (*211*)	2.12	7.58 ± 0.02	25	26	21.8
U1383C Middle	146–200	3.6	n.d.	2.23	7.58 ± 0.03	27	26	21.9
U1383C Deep	200–332	3.8	213 ± 1 (*2370*)	2.26	7.60 ± 0.01	23	24	21.8
Seawater	~4400 m	n.d.	~240	~2.02*	7.53 ± 0.01	50	25	21.1

Oxygen measurements were made *in situ* via optode, while total CO_2_ (tCO_2_) estimates were made onboard *via* mass spectrometry.

n.d. = not determined

* = Sargasso seawater tCO_2_ as in Bates *et al*.^68^.

**Table 2:** Concentration of select trace elements in North Pond subsurface crustal fluids and the overlying deep Atlantic bottom seawater.

Sample	Li (μmol kg^−1^)	B (μmol kg^−1^)	Na (mmol kg^−1^)	Mg (mmol kg^−1^)	Si (μmol kg^−1^)	S (mmol kg^−1^)	K (mmol kg^−1^)	Ca (mmol kg^−1^	V (nmol kg^−1^)	Rb (nmol kg^−1^)	Sr (μmol kg^−1^)	Mo (nmol kg^−1^)	Cs (nmol kg^−1^)	Ba (nmol kg^−1^)	U (nmol kg^−1^)
U1382A	26.3	407	461	52.1	56	27.8	10.0	10.1	22	1290	89.9	120	2.46	129	15.0
U1383C Shallow	26.3	409	456	51.8	125	27.4	9.9	10.0	44	1330	89.6	120	2.38	128	13.4
U1383C Middle	25.8	399	454	51.0	123	27.0	9.7	9.8	42	1290	88.5	120	2.38	113	13.0
U1383C Deep	26.3	406	459	52.4	120	27.6	10.0	10.1	42	1305	89.6	124	2.39	120	13.9
Seawater	26.5	412	461	52.4	48	27.8	10.0	10.1	31	1320	90.6	120	2.07	82	15.2

Mn and Fe were below the detection limit (<0.1 μmol kg^−1^) in all samples.

**Table 3:** Carbon and nitrogen content of particulates in North Pond subsurface crustal fluids and the overlying deep Atlantic bottom seawater.

	POC (μmol L^−1^)	PN (μmol L^−1^)	POC/TN (molar ratio)
U1382A	1.2	0.02	62
U1383C Shallow	0.8	0.02	55
U1383C Middle	0.4	0.01	27
U1383C Deep	1.2	0.03	42
Seawater	0.3	0.05	7
Seawater	0.1	0.02	8

**Table 4:** Total microbial cell densities, as well as bacterial and 16S rRNA gene abundance, in crustal fluids, bottom seawater, and drilling mud.

	Cells ml^−1^ of fluid (±95% confidence level)	Relative abundance of cells hybridized with bacteria-specific probe EUB33 (%)	Average 16S rRNA gene copies per ng extracted DNA (±std dev)
U1382A	1.4 × 10^4^ (±6 × 10^2^)	93 ± 2	3.9 × 10^3^ (±9 × 10^2^)
U1383C Shallow	2.0 × 10^4^ (±7 × 10^2^)	97 ± 2	4.9 × 10^2^ (±6 × 10^1^) *in situ*
			5.0 × 10^3^ (±9 × 10^2^)
U1383C Middle	2.2 × 10^4^ (±8 × 10^2^)	98 ± 2	1.2 × 10^3^ (±3 × 10^1^) *in situ*
U1383C Deep	2.1 × 10^4^ (±8 × 10^2^)	97 ± 2	3.6 × 10^2^ (±1 × 10^2^) *in situ*
			6.0 × 10^2^ (±8 × 10^1^)
Seawater	2.2 × 10^4^ (±5 × 10^2^)	91 ± 3	7.5 × 10^3^ (±6 × 10^3^)
Drilling mud	n.d.	n.d.	2.6 × 10^2^ (±2 × 10^2^)

DNA was extracted from filters preserved after retrieval on board ship or preserved on the seafloor (samples designated “*in situ*”). n.d. = not determined.
