# Author Correction: A distinct and active bacterial community in cold oxygenated fluids circulating beneath the western flank of the Mid-Atlantic ridge

**DOI:** 10.1038/s41598-020-63127-z

**Published:** 2020-04-16

**Authors:** Julie L. Meyer, Ulrike Jaekel, Benjamin J. Tully, Brian T. Glazer, C. Geoffrey Wheat, Huei-Ting Lin, Chih-Chiang Hsieh, James P. Cowen, Samuel M. Hulme, Peter R. Girguis, Julie A. Huber

**Affiliations:** 1 Josephine Bay Paul Center, Marine Biological Laboratory, 7 MBL St., Woods Hole, MA 02543 USA; 2 Department of Organismic and Evolutionary Biology, Harvard University, 16 Divinity Ave., Cambridge, MA 02138 USA; 3 Center for Dark Energy Biosphere Investigations, University of Southern California, 3616 Trousdale Parkway, Los Angeles, CA 90089 USA; 4 Department of Oceanography, University of Hawai’i at Mānoa, 1000 Pope Rd, Honolulu, HI 96822 USA; 5 Global Undersea Research Unit, University of Alaska Fairbanks, P.O. Box 475, Moss Landing, CA 95039 USA; 6 Moss Landing Marine Laboratory, 8272 Moss Landing Road, Moss Landing, CA 95039 USA; 7 Present Address: Soil and Water Science Department, University of Florida, 2033 Mowry Rd, Gainesville, FL 32606 USA; 8 Present Address: Shell Technology Norway AS, Karenslyst Alle 2, N-0277 Oslo, Norway

Correction to: *Scientific Reports* 10.1038/srep22541, published online 03 March 2016

This Article contains an error in Table 1. For the Oxygen concentration of the seawater sample, “~240” should read “~250”.

