# Supplementary material for: A distinct and active bacterial community in cold oxygenated fluids circulating beneath the western flank of the Mid-Atlantic ridge
Source: Sci Rep. 2016 Mar 3;6:22541. doi: 10.1038/srep22541 (PMC4776111; doi:10.1038/srep22541)
Supplement: Supplementary Information [file srep22541-s1.pdf]

SUPPLEMENTARY METHODS and RESULTS for

A distinct and active bacterial community in cold oxygenated fluids circulating beneath the western flank of the Mid-Atlantic ridge

Julie L Meyer[a,1,2], Ulrike Jaekel[b,1,3], Benjamin Tully[c], Brian T Glazer[d], C Geoffrey Wheat[e], Huei-Ting Lin[d], Chih-Chiang Hsieh[d], James P. Cowen[d], Samuel M Hulme[f], Peter R Girguis[b], and Julie A Huber[a,4]

Author affiliations:
[a]Josephine Bay Paul Center, Marine Biological Laboratory, 7 MBL St., Woods Hole, MA 02543
[b]Department of Organismic and Evolutionary Biology, Harvard University, 16 Divinity Ave., Cambridge, MA 02138
[c]Center for Dark Energy Biosphere Investigations, University of Southern California, 3616 Trousdale Parkway, Los Angeles, CA 90089
[d]Department of Oceanography, University of Hawai'i at Mānoa, 1000 Pope Rd., Honolulu, HI 96822
[e]Global Undersea Research Unit, University of Alaska Fairbanks, P.O. Box 475, Moss Landing, CA 95039
[f]Moss Landing Marine Laboratory, 8272 Moss Landing Road, Moss Landing, CA 95039

Author notes:
[1]Shared first-authorship.
[2]Current address: Soil and Water Science Department, University of Florida, 2033 Mowry Rd., Gainesville, FL, 32606
[3]Current address: Shell Technology Norway AS, Karenslyst Alle 2, N-0277 Oslo, Norway
[4]Author for correspondence:

Dr. Julie A Huber
jhuber@mbl.edu
phone: 508-289-7291
fax: 508-457-4727

Metagenomic Sequence Analysis

Sequences were assessed for Illumina adapter sequences in the 3'-end of the read using cutadapt v1.7.1 (parameters: -a AGATCGGAAGAGC -e 0.08 –overlap=3) [1]. Sequences were then trimmed based on quality scores using Trimmomactic v0.33 with a sliding window of 10bp and an average quality score cutoff of 28 [2]. All sequences trimmed below 75bp in length were discarded (parameters: SLIDINGWINDOW:10:28 MINLEN:75). Only read pairs for which both mate survived trimming were retained for assembly and read coverage analysis. For each sample library, quality trimmed sequences were assembled using IDBA-UD v1.1.1 using the default parameters [3]. Contigs < 500bp in length were removed from consideration for further analysis.

Assemblies and putative CDS were retrieved for samples from the Guaymas (Taxon object ID: 3300001683) and Abe, Lau Basin (Taxon object ID: 3300001681) hydrothermal vent plumes. Both sets of assemblies were generated using IDBA. Assemblies from sediment sampled at 5 cmbsf from the south Pacific (unpublished) and at 75 cmbsf from the Arctic Mid-Ocean ridge (DDBJ/EMBL/Genbank Accession: LAZR00000000) 4 were processed using Prodigal to generate putative CDS. The sample from the south Pacific was assembled using IDBA-UD. The sample from the Arctic Mid-Ocean ridge was assembled using SPAdes v3.0.0 [5].

Putative CDS for the NP samples and the additional metagenomes were searched using HMMER3 v3.1b1 [6] against the TIGRFAM v14 database [7] (hmmsearch, parameters: -E 0.00001). From the hmmsearch results, putative CDS were assigned to TIGRFAM roles based on the best match. For each metagenome, the relative abundance of each TIGRFAM role was determined (no. of putative CDS assigned to a specific role ÷ total no. of putative CDS assigned to all TIGRFAM roles). The relative abundance of the 115 identified TIGRFAM roles for each sample was visualized using principal component analysis (PCA) to determine the relationship between samples. PCA was performed using the Python library sklearn v0.16.1. Values underwent dimensionality reduction, while being fit to the model.

Assessment of Carbon Fixation Potential

Utilizing the IMG annotations, a database of genes representing the necessary and essential components of the major carbon fixation pathways searched using BLASTp v2.2.30+

(parameters: -evalue 0.001 -max_target_seqs 3) against the putative CDS for each NP sample [8]. The database contained genes necessary to identify the reductive acetyl-CoA/Wood-Ljunghal pathway, the reverse citric acid (rTCA) cycle, the Calvin-Bensson-Bassham (CBB) cycle, the 3-hydroxypropionate/4-hydroxybutyrate (3-OH-propionate/4-OH-butryrate) cycle, and the 3-hydroxypropionate (3-OH-propionate) bicycle. Successful matches were limited to matches with > 30% amino acid identity (AAID) and > 30% protein alignment. Putative carbon fixation relevant CDS were used to recruit sequences from quality trimmed sequence libraries, which had been normalized by random sampling to the size of the smallest library (27,737,142 sequences), using BWA v0.6.1-r104 (parameters: bwa aln -n 0; bwa samse -n 0) [9]. Utilizing the output SAM file, the number of sequences assigned to a specific carbon fixation gene was determined.

The quality trimmed metagenomes were queried using Meta-RNA (parameters: -m ssu -e 1e-10) to identify putative 16S rRNA gene fragments [10]. Putative 16S rRNA fragments were then assembled using EMIRGE [11] using the SILVA SSURef_111 [12] as a guide (emirge_amplicon.py, parameters: -l 113 -i 163 -s 33 -a 32 –phred33). As part of the EMIRGE assembly process, a length-normalized estimate of relative abundance was determined and full length 16S rRNA sequences with > 1% estimated abundance were identified.

Putative taxonomies were assigned to the assembled full length 16S rRNA sequences using mothur v1.34.4 [13]. 16S rRNA sequences were aligned to a SILVA SSURef111 database containing sequences from all three domains of life (align.seqs). Sequences that failed to align in this step were removed (remove.seqs). The remaining sequences were classified based on the SILVA taxonomy (classify.seqs, parameters: cutoff=80, iters=1000).

To determine the putative taxonomies of certain carbon fixation processes (rTCA and Calvin cycles), the putative carbon fixation CDS were BLASTp (parameters: -evalue 1e-5 -max_target_seqs 5) against the NCBI RefSeq database [14]. MEGAN4 was then used to determine the last common ancestor (LCA) of the top five hits for each putative carbon fixation CDS [15]. Utilizing the number of sequences recruited to each putative carbon fixation CDS, CDS with the same taxonomic assignment, up to the Genus level, had their sequence counts combined and was compared to the total number of sequences recruited for that gene.

For the 100 essential phylogenetic markers gene analysis, putative CDS identified were BLASTp against the NCBI RefSeq database (as above). MEGAN4 was used to assign an LCA to each putative phylogenetic marker based on the top five best RefSeq matches, as described in [16]. Each putative phylogenetic marker was assigned taxonomy at the Class level. Putative markers without assignments at the Class level were consider to lack a taxonomic assignment, though many possess assignments at higher taxonomic levels. The percent relative abundance of each putative phylogenetic marker within the NP metagenomes was determined using libraries normalized by random subsampling to the size of the smallest metagenome and were recruited to the putative phylogenetic markers using BWA (as above). The number of recruited sequences was determined from the SAM file and the sequence count of putative marker genes with the same taxonomic assignment were combined (no. of sequences assigned to putative phylogenetic markers within a Class ÷ total no. of sequences recruited to all putative phylogenetic markers [including those without assignments] × 100).

Metagenomic Results

A total of 29.9 Gbp sequence was generated, with an average of 7.5 ± 1.1 Gbp per sample. Following the quality control step, 21.6 Gbp of sequence was retained for assembly, with an average of 5.4 ± 1.6 Gbp per sample. The remaining number of paired-end sequences ranged from 27-57 million sequences. After excluding contigs < 500 bp in length, a total of 458,479 contigs were generated for all samples, with 114,620 ± 66,526 contigs per sample. These contigs represent a total of 637 Mbp of assemblies, with 159 ± 105 Mbp per sample. For the collective set of contigs, the mean contig length was 1,429 bp (N50 = 1,878 bp).

The number of putative CDS predicted by Prodigal was on average ± 2.3% of the number of putative CDS predicted using the IMG annotation pipeline. In total, the IMG pipeline predicted 937,375 putative CDS from the North Pond metagenomes, with 234,343 ± 146,386 putative CDS per sample.

REFERENCES

1 Martin, M. Cutadapt removes adapter sequences from high-throughput sequencing reads. *EMBnet.journal* **17**, doi:http://dx.doi.org/10.14806/ej.17.1.200 (2011).
2 Bolger, A. M., Lohse, M. & Usadel, B. Trimmomatic: a flexible trimmer for Illumina sequence data. *Bioinformatics* **30**, 2114-2120 (2014).
3 Peng, Y., Leung, H. C. M., Yiu, S. M. & Chin, F. Y. L. IDBA-UD: a de novo assembler for single-cell and metagenomic sequencing data with highly uneven depth. *Bioinformatics* **28**, 1420-1428, doi:Doi 10.1093/Bioinformatics/Bts174 (2012).
4 Spang, A. *et al.* Complex archaea that bridge the gap between prokaryotes and eukaryotes. *Nature* **521**, 173-179 (2015).
5 Bankevich, A. *et al.* SPAdes: A new genome assembly algorithm and its application to single-cell sequencing. *Journal of Computational Biology* **19**, 455-477 (2012).
6 Finn, R. D., Clements, J. & Eddy, S. R. HMMER web server: interactive sequence similarity searching. *Nucleic Acids Res* **39**, W29-W37 (2011).
7 Haft, D. H. The TIGRFAMs database of protein families. *Nucleic Acids Res* **31**, 371-373 (2003).
8 Altschul, S. F., Gish, W., Miller, W., Myers, E. W. & Lipman, D. J. Basic Local Alignment Search Tool. *Journal of Molecular Biology* **215**, 403-410 (1990).
9 Li, H. & Durbin, R. Fast and accurate short read alignment with Burrows-Wheeler transform. *Bioinformatics* **25**, 1754-1760 (2009).
10 Huang, Y., Gilna, P. & Li, W. Identification of ribosomal RNA genes in metagenomic fragments. *Bioinformatics* **25**, 1338-1340 (2009).
11 Miller, C. S., Baker, B. J., Thomas, B. C., Singer, S. W. & Banfield, J. F. EMIRGE: reconstruction of full-length ribosomal genes from microbial community short read sequencing data. *Genome Biology* **12**, R44 (2011).
12 Quast, C. *et al.* The SILVA ribosomal RNA gene database project: Improved data processing and web-based tools. *Nucleic Acids Res* **41**, 590-596, doi:10.1093/nar/gks1219 (2013).
13 Schloss, P. D. *et al.* Introducing mothur: Open-source, platform-independent, community-supported software for describing and comparing microbial communities. *Applied and Environmental Microbiology* **75**, 7537-7541 (2009).
14 Tatusova, T., Ciufo, S., Fedorov, B., O'Neill, K. & Tolstoy, I. RefSeq microbial genomes database: New representation and annotation strategy. *Nucleic Acids Res* **43**, 3872-3872 (2015).
15 Huson, D. H., Mitra, S., Ruscheweyh, H.-J., Weber, N. & Schuster, S. C. Integrative analysis of environmental sequences using MEGAN4. *Genome Research* **21**, 1552-1560 (2011).
16 Albertsen, M. *et al.* Genome sequences of rare uncultured bacteria obtained by differential coverage binning of multiple metagenomes. *Nature Biotechnology* **31**, 533-538 (2013).

SUPPLEMENTARY TABLES AND FIGURE LEGENDS

Supplementary Table 1 A. *Raw data and calculated rates for incubations with ^{13}C-labelled bicarbonate (Autotrophy).* The time points used for rate calculations* were determined by plotting all time points against the corresponding Delta ^{13}Cvpdb values (not shown) and then choosing only those values that make up the exponential phase of the resulting curve.

Sample origin	Days of incubation at 5°C	Delta ^{13}Cvpdb at 5°C	Rate (pmol C ml^{-1} d^{-1}) at 5°C	Days of incubation at 25°C	Delta ^{13}Cvpdb at 25°C	Rate (pmol C ml^{-1} d^{-1}) at 25°C
Deep Seawater	0<br>3*<br>11*<br>13*<br>74	-27.89<br>-26.85<br>-25.94<br>-20.52<br>-6.90	856	0<br>3*<br>11*<br>13*<br>21	-27.89<br>-11.74<br>-2.40<br>-1.93<br>8.17	1334
U1382A	0<br>2*<br>8*<br>10*<br>49	-28.46<br>-26.63<br>-20.70<br>-18.75<br>27.65	1332	0*<br>2*<br>10*<br>28<br>49	-27.26<br>-19.47<br>0.24<br>1.31<br>2.10	3742
U1383C-Shallow	0*<br>2*<br>10*<br>24<br>45	-27.02<br>-13.27<br>-4.71<br>-2.96<br>-3.30	3031	0<br>2*<br>8*<br>10*<br>-	-27.64<br>-25.19<br>-23.83<br>-5.89<br>-	3277
U1383C-Deep	0*<br>2*<br>7*<br>44<br>70	-27.00<br>-26.69<br>-25.33<br>4.46<br>5.08	322	0*<br>2*<br>9*<br>38<br>74	-27.78<br>-20.50<br>0.84<br>-4.01<br>6.68	4328

Supplementary Table 1 B. *Raw data and calculated rates for incubations with ^{13}C-labelled acetate (Heterotrophy)***.** The time points used for rate calculations* were determined by plotting all time points against the corresponding Delta ^{13}Cvpdb values (not shown) and then choosing only those values that make up the exponential phase of the resulting curve.

Sample origin	Days of incubation at 5°C	Delta ^{13}Cvpdb at 5°C	Rate (pmol C ml^{-1} d^{-1}) at 5°C	Days of incubation at 25°C	Delta ^{13}Cvpdb at 25°C	Rate (pmol C ml^{-1} d^{-1}) at 25°C
Deep Seawater	0<br>3*<br>11*<br>13*<br>74	-25.79<br>-25.61<br>7.72<br>19.4<br>86.26	25.9	0<br>3*<br>11*<br>13*<br>-	-26.13<br>86.67<br>117.54<br>135.16<br>-	28.3
U1382A	0*<br>2*<br>8*<br>10<br>49	-24.37<br>-10.12<br>56.56<br>30.3<br>49.42	58.4	0*<br>2*<br>10*<br>28<br>49	-24.96<br>75.2<br>153.36<br>74.81<br>91.26	104.3
U1383C-Shallow	0<br>2*<br>10*<br>23*<br>45	-25.66<br>239.36<br>136.47<br>174.98<br>114.29	94.6	0<br>2*<br>8*<br>10*<br>45	-25.32<br>-23.83<br>-14.75<br>96.4<br>80.54	87.3
U1383C-Deep	0*<br>2*<br>7*<br>70<br>-	-25.71<br>-15.00<br>-18.17<br>80.14<br>-	6.2	0*<br>2*<br>9*<br>38<br>74	-25.42<br>42.13<br>81.47<br>72.52<br>199.97	68.8

Supplementary Table 2. Relative abundance of 16S rRNA and 16S rRNA gene amplicon reads assigned to taxa that occurred less than 1% in any individual sample of crustal fluids from Holes U1382A and U1383C, deep Atlantic bottom seawater, and drilling mud.

	1382A		1383C Shallow				1383C Middle		1383C Deep				Seawater		Mud
	DNA	RNA	DNA	DNA	RNA	RNA	DNA	RNA	DNA	DNA	RNA	RNA	DNA	RNA	DNA
**Archaea**	0.0592%	0.0027%	0.0243%	0.1084%	0.0022%	0.0013%	0.1315%	0.0007%	0.1155%		0.0001%	0.0012%	0.0140%	0.0061%	0.0279%
**Armatimonadetes**		0.0020%			0.0002%			0.0005%						0.0016%	
**BD1-5**		0.0011%	0.0012%	0.1139%	0.0010%	0.0013%		0.0025%			0.0007%	0.0017%	0.0134%	0.0079%	0.0186%
**BHI80-139**		0.0001%			0.0001%	0.0001%		0.0001%							
**Cand div BRC1**		0.0091%			0.0237%	0.0465%		0.0409%			0.0201%	0.0313%		0.2545%	0.1826%
**Cand div OD1**	0.0878%	0.0165%	0.2090%		0.1790%	0.3108%	0.8956%	0.1936%	0.0615%		0.0748%	0.2065%		0.0046%	0.0124%
**Cand Div OP3**	0.2391%	0.1229%			0.0036%	0.0031%		0.0037%			0.0014%	0.0032%		0.0091%	
**Cand Div OP11**	0.0019%	0.0019%			0.0058%	0.0070%		0.0029%				0.0014%			
**Cand Div OP9**					0.0001%			0.0002%							
**Cand Div TM9**		0.0009%	0.0012%		0.0001%	0.0003%		0.0001%		0.1516%			0.0268%		
**Cand Div WS3**		0.0012%			0.0005%	0.0020%		0.0010%			0.0001%	0.0003%		0.0013%	
**Cand Div WS6**						0.0003%					0.0001%			0.0030%	
**Chlamydiae**		0.0033%	0.0693%		0.0086%	0.0108%	0.1216%	0.0091%			0.0140%	0.0035%		0.0282%	0.1114%
**Chlorobi**		0.0041%			0.0006%	0.0006%	0.0037%	0.0015%			0.0008%	0.0009%		0.0143%	
**Deinococcus-Thermus**		0.0007%	0.0211%		0.0003%	0.0012%		0.0039%			0.0009%	0.0012%		0.0129%	0.0248%
**Dictyoglomi**												0.0006%		0.0001%	
**Elusimicrobia**		0.0167%			0.0013%	0.0016%		0.0030%			0.0007%	0.0003%		0.0003%	
**Fibrobacteres**		0.0072%	0.0552%		0.0010%	0.0015%		0.0064%			0.0031%	0.0075%		0.0051%	0.1238%
**Fusobacteria**	0.0427%	0.0219%	0.1304%		0.0003%	0.0009%	0.0381%	0.0023%			0.0003%			0.0042%	
**Hyd24-12**		0.0001%												0.0001%	
**JL-ETNP-Z39**		0.0004%													
**Lentisphaerae**		0.0682%			0.0697%	0.0989%		0.1931%	0.0390%		0.1311%	0.0828%	0.2398%	0.7611%	
**MVP-21**		0.0002%										0.0006%			
**Nitrospirae**	0.0146%	0.0352%		0.8245%	0.0107%	0.0179%		0.0086%			0.0106%	0.0104%		0.2634%	
**Other Proteobacteria**		0.0337%	0.0160%	0.0508%	0.0892%	0.1155%	0.0860%	0.0447%	0.0015%		0.1093%	0.1297%	0.4871%	0.2557%	0.0371%
**NPL-UPA2**	0.0189%	0.0368%			0.0060%	0.0060%		0.0041%			0.0035%	0.0035%		0.1441%	
**Spirochaetes**		0.0296%	0.0196%		0.0060%	0.0179%	0.1990%	0.0272%			0.0148%	0.0227%	0.0012%	0.3357%	
**Synergistetes**		0.0010%			0.0002%			0.0003%			0.0002%	0.0003%		0.0003%	
**TA06**		0.0015%			0.0002%	0.0006%		0.0008%			0.0006%	0.0012%		0.0028%	
**Tenericutes**									0.0435%					0.0005%	
**Thermotogae**		0.0006%			0.0003%			0.0002%							
**TM6**		0.0334%			0.1052%	0.0934%		0.0981%			0.0598%	0.0722%		0.0049%	
**WCHB1-60**					0.0001%									0.0004%	0.0155%
**Eukaryota**	0.0029%	0.0006%	0.0012%	0.0604%	0.0003%	0.0004%	0.0111%	0.0001%	0.0330%	0.0437%	0.0015%	0.0003%	0.0018%		

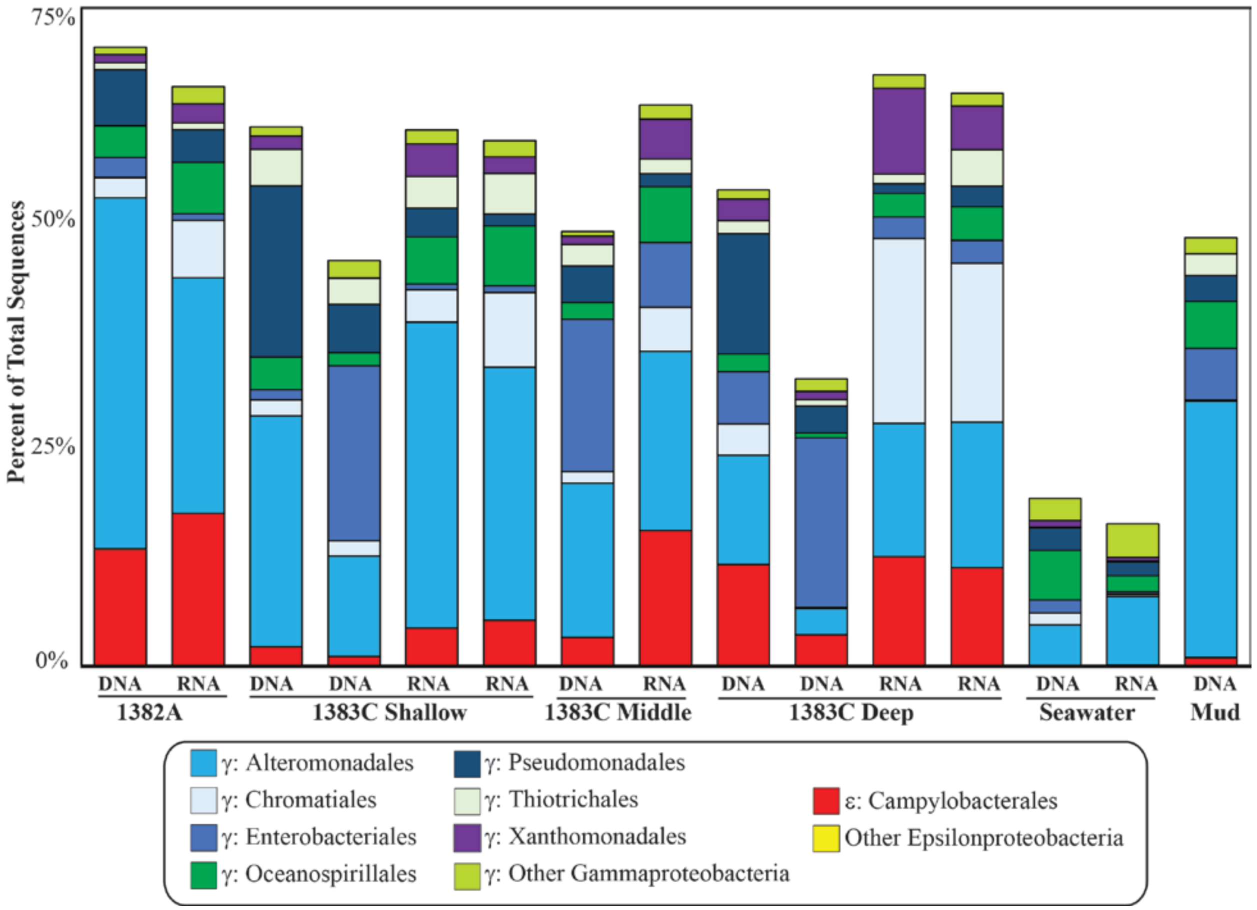

Supplementary Figure 1. Relative abundance of 16S rRNA and 16S rRNA gene V6 amplicon reads assigned to *Gammaproteobacteria* and *Epsilonproteobacteria* at the Order level in crustal fluids from Holes U1382A and U1383C, deep Atlantic bottom seawater, and drilling mud.

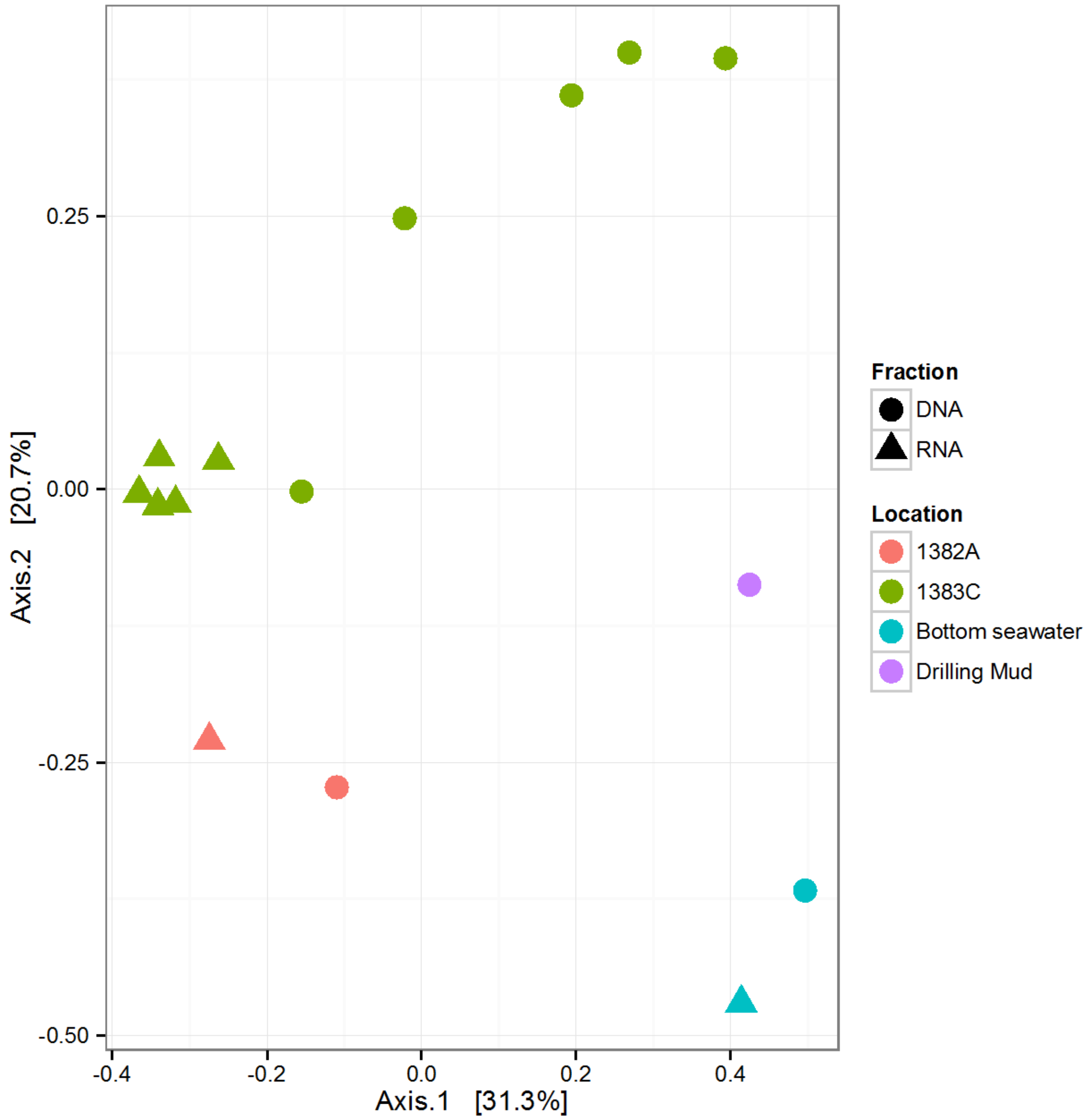

Supplementary Figure 2. Bacterial community similarity based on Morisita-Horn beta diversity of 16S rRNA and 16S rRNA gene V6 amplicon data from North Pond crustal fluids from U1382A and U1383C, in bottom seawater and in a sample of drilling mud from the installation of the CORK observatories.

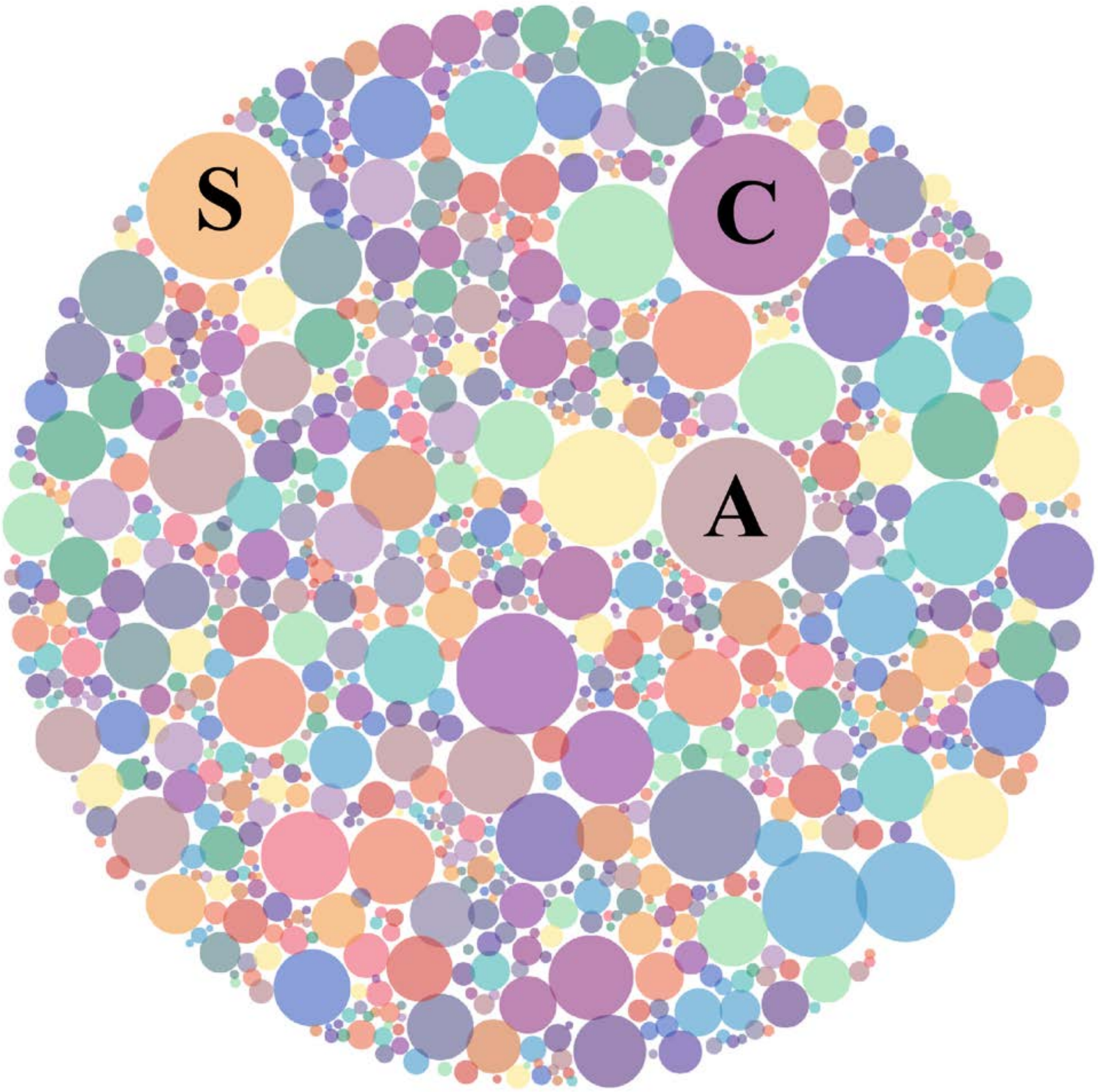

Supplementary Figure 3. Abundant genera in North Pond crustal fluids based on 16S rRNA and 16S rRNA gene V6 amplicon data showing total relative abundance of *Colwellia* ("C"), *Sulfurimonas* ("S"), and *Acidiferrobacter* ("A") out of 1,023 genera in all crustal fluid samples.

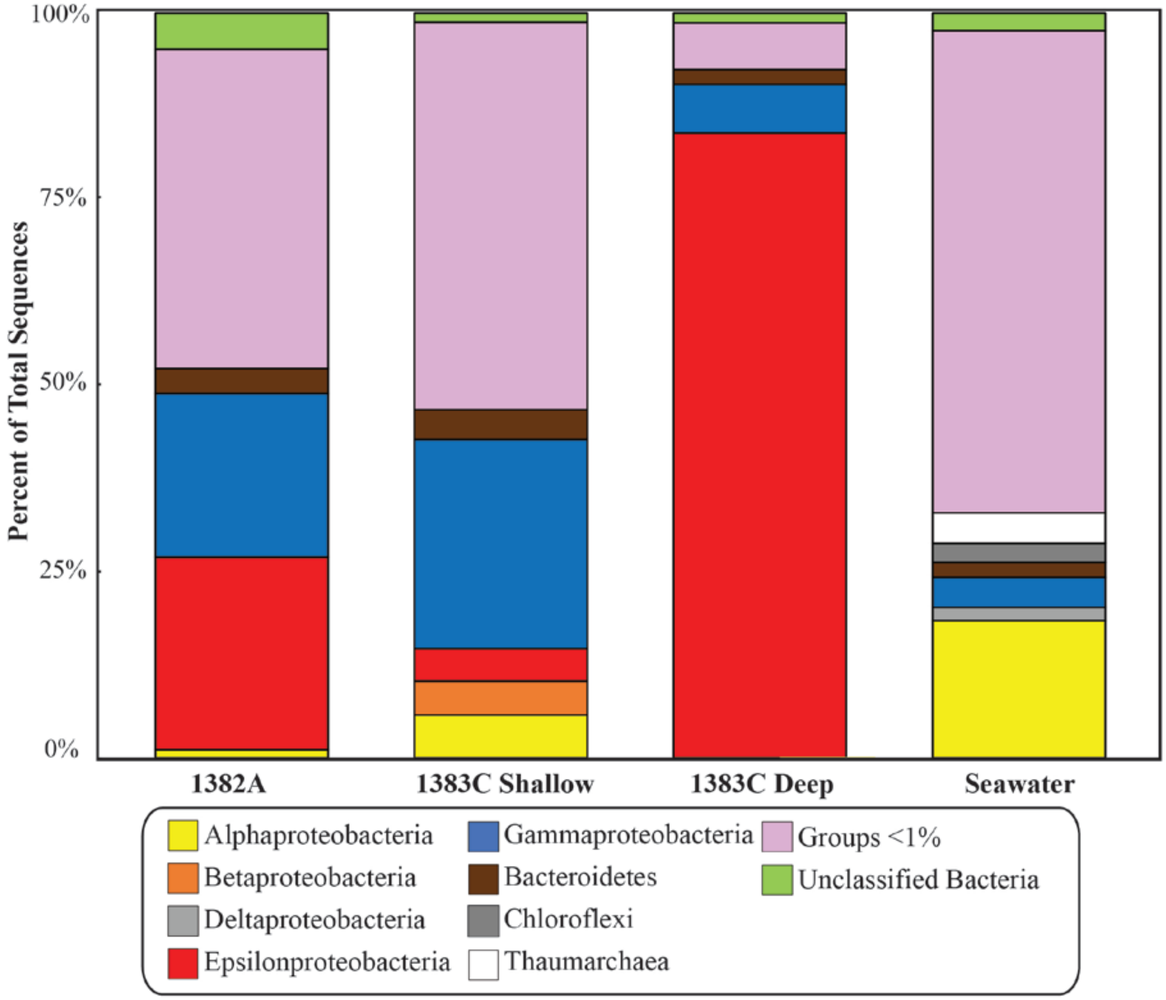

Supplementary Figure 4. Relative abundance and taxonomic assignment of full-length 16S rRNA genes as reconstructed from North Pond metagenomes.

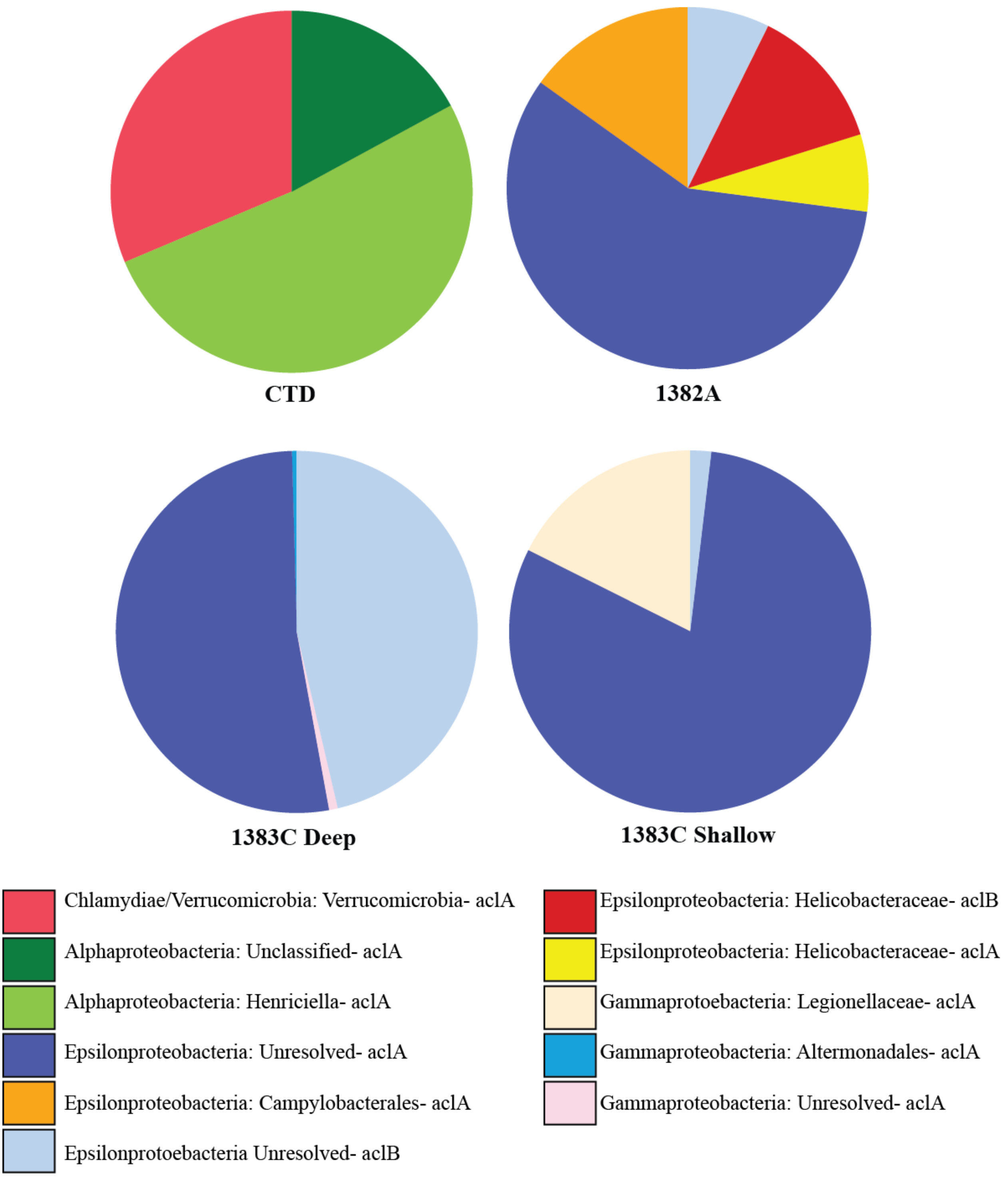

Supplementary Figure 5. Taxonomic assignment of *aclA* and *aclB* from North Pond metagenomes.

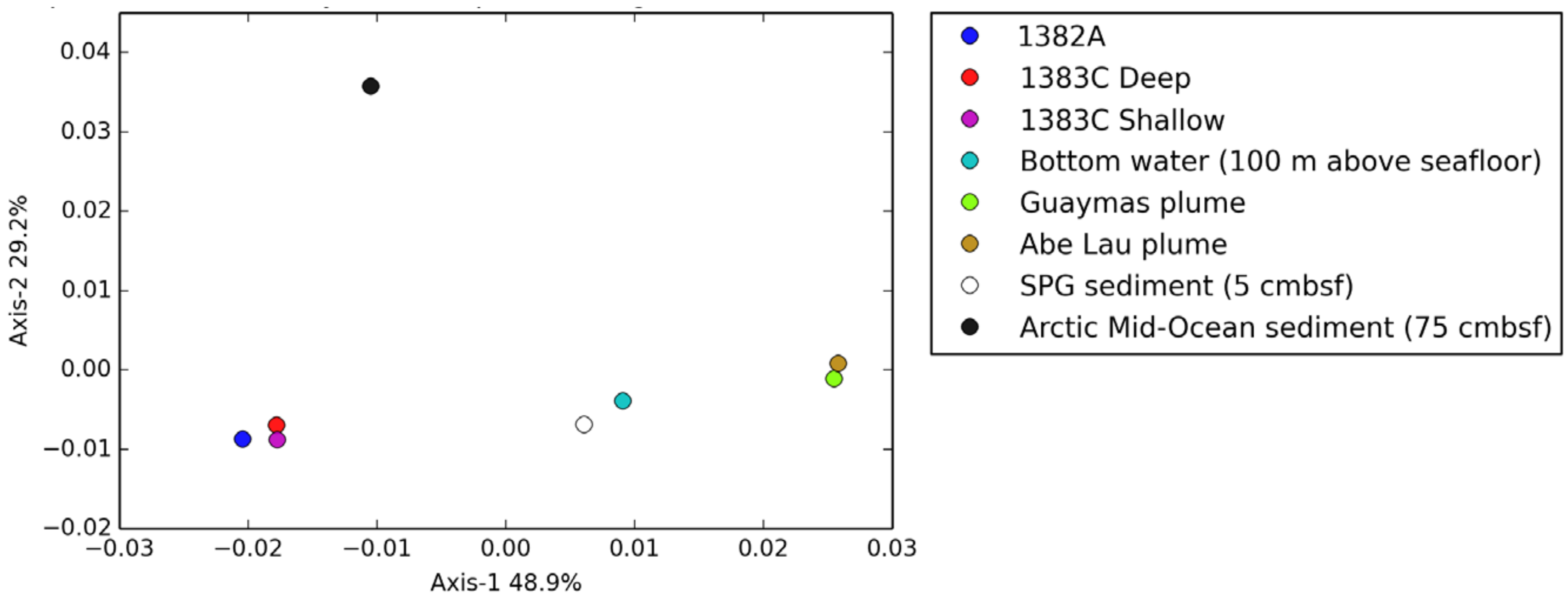

Supplementary Figure 6. Principle Coordinate Analysis of deep-sea metagenomes based on TIGRFAM abundance. Bottom water is the CTD sample collected 100 meters off the seafloor as part of this study.
